# Assessment of recall error in self-reported food consumption histories among adults—Particularly delay of interviews decrease completeness of food histories—Germany, 2013

**DOI:** 10.1371/journal.pone.0179121

**Published:** 2017-06-22

**Authors:** Maximilian Gertler, Irina Czogiel, Klaus Stark, Hendrik Wilking

**Affiliations:** 1 Department for Infectious Disease Epidemiology, Robert Koch Institute (RKI), Berlin, Germany; 2 Postgraduate Training for Applied Epidemiology (PAE), Robert Koch Institute (RKI), Berlin, Germany; 3 European Programme for Intervention Epidemiology Training, ECDC, Stockholm, Sweden; Australian National University, AUSTRALIA

## Abstract

**Introduction:**

Poor recall during investigations of foodborne outbreaks may lead to misclassifications in exposure ascertainment. We conducted a simulation study to assess the frequency and determinants of recall errors.

**Methods:**

Lunch visitors in a cafeteria using exclusively cashless payment reported their consumption of 13 food servings available daily in the three preceding weeks using a self-administered paper-questionnaire. We validated this information using electronic payment information. We calculated associated factors on misclassification of recall according to time, age, sex, education level, dietary habits and type of servings.

**Results:**

We included 145/226 (64%) respondents who reported 27,095 consumed food items. Sensitivity of recall was 73%, specificity 96%. In multivariable analysis, for each additional day of recall period, the adjusted chance for false-negative recall increased by 8% (OR: 1.1;95%-CI: 1.06, 1.1), for false-positive recall by 3% (OR: 1.03;95%-CI: 1.02, 1.05), for indecisive recall by 12% (OR: 1.1;95%-CI: 1.08, 1.15). Sex and education-level had minor effects.

**Discussion:**

Forgetting to report consumed foods is more frequent than reporting food-items actually not consumed. Bad recall is strongly enhanced by delay of interviews and may make hypothesis generation and testing very challenging. Side dishes are more easily missed than main courses. If available, electronic payment data can improve food-history information.

## Introduction

Interviewing sick persons concerning their food history is probably the oldest and most important method used for hypothesis generation in investigations of outbreaks of foodborne infectious disease. In a next step, analytical studies comparing interview data from sick and healthy people (case control or cohort design) allows for hypothesis testing. This strategy is recommended by international guidelines [1–4]. Interviewees´ poor recall can lead to exposure misclassification of food items which is a frequent experience of any public health epidemiologist which can lead to problems in identifying and testing hypotheses. Misclassification may hinder identification of contaminated vehicles in food-borne outbreaks [5]. If a vehicle is poorly remembered it can hardly be detected. At the same time, uncontaminated food items which are associated with the actual vehicle but better recalled could be wrongly suspected. This is especially problematic for outbreaks of diseases with long incubation periods including listeriosis, Hepatitis A or during the outbreak of Shiga toxin–producing Escherichia coli (STEC) O104:H4 infection in Germany 2011 [6]. Additionally, interview-based investigations are even more difficult when the disease sets patients into a state in which they cannot be interviewed.

During the STEC outbreak in 2011 in Germany, studies designed independently from the human recall capability have been particularly successful [7]. In one of the case-control studies, the cashless payment system of a company cafeteria used by the investigators provided food histories of patients and controls in a short time [8]. Other similar experiences of use of electronic payment data to investigate foodborne outbreaks were reported [9–11].

Little information is available about the actual frequency and determinants of recall error and misclassification of food items. In a study from 1986 epidemiologists investigated food recall during a luncheon in their institute. The investigators videotaped 32 attendees at the buffet table and interviewed them afterwards concerning their food selection. Consumers failed more often to report selection of desserts and bread compared to other servings, but influence of recall period could not be studied [12]. Similarly, Mann et al. observed attendees of a luncheon documenting their selection. Then, they compared the observed food choice with reported food history of the attendees from questionnaire-based interviews five days after the meal. They estimated sensitivity of recall of 88% and specificity between 73% and 93% [5].

To better understand determinants of food history recall, we simulated an outbreak investigation and used electronic data from personal payment cards as gold standard for food history in a cafeteria in Berlin, Germany, to check the recall of the consumers as ascertained using a paper-based questionnaire.

## Material and methods

Visitors of a company cafeteria in a bank in Berlin were approached and interviewed during the regular opening hours at lunchtime (11:45 AM to 2:30 PM). In the morning of the same day, all employees with access to the cafeteria received an information letter via email, informing them about the interviews, the simulative and anonymous nature of the study. In the cafeteria, employees of the Robert Koch Institute (RKI), the responsible public health agency for the control of infectious diseases in Germany, approached the cafeteria guests to further inform them about the study and invite them to participate.

Participants were asked to fill in a standardized questionnaire about daily cafeteria visits and their food consumption of 13 different regularly served items in the cafeteria during the preceding three weeks (15 opening days). Additionally, personal characteristics (year of birth, sex, education degree), information on dietary habits (eating vegetarian, low-calorie-diet and having any food intolerance) and the personal customer identifier code number (ID) displayed on the card of the cashless payment system were retrieved. The questionnaire was designed using the same layout as the normal weekly menu of the cafeteria to increase ability to remember as might have been done by the field epidemiology team in a real outbreak scenario. Every day, the canteen offers three different main courses which, like four of the five offered side dishes and like two of the three desserts, vary every day. In addition, consumers may choose from a salad bar and may choose to take bakery (a roll or a bread) with their lunch. For analysis, we grouped the varying categories together, into 8 food item categories: main courses, side dishes, boiled potatoes (the non-varying third side dish), vegetable side dishes, desserts, fruit-salad (the non-varying third dessert), salad-bar (available every day) and bakery (available every day). To visualise the questionnaire, it is provided in supportive information files “S1 Questionnaire German” and “S1 Questionnaire English“.

The management of the cafeteria provided printed copies of the canteen payment of each participant’s IDs. All paper records were digitalised with software EpiData Entry (http://www.epidata.dk/). Double data entry and checks were performed for all data to reduce data entry errors.

For analysis the electronic payment information was used as standard and misclassifications were categorised as false-positive (reported eaten, not paid), false-negative (reported not eaten, but paid) and indecisive (Don’t know-answer). We used multivariable logistic regression separately for each misclassification category as dependent variable. We used recall period, sex, age group, degree of education, dietary habits and food item categories in each model as independent variables, without selection of variables. Statistical analysis was performed with STATA version 12.1C.

In this study, anonymous data on food histories and demographic characteristics were retrieved. No information on disease, disease-related states or disease-relevant exposures were collected. In detail, we asked healthy volunteers to report anonymously about their food intake in their canteen—there was no outbreak, nobody was asked for symptoms or about his/her medical condition, nobody was treated or underwent biomedical diagnostic tests or similar.

Participants were informed before and at the beginning of the survey about the simulation character of the study and were only included after written informed consent. We compared the reported food histories with those registered by the electronic payment system (identification by canteen card ID number).

To guarantee the highest possible level of anonymity, we requested and received approval of the data safety office at the Robert Koch Institute (the German National Public Health Institute). Therefore we consider this study to be in accordance with the Declaration of Helsinki without having applied for a review of an institutional ethics committee prior to the interviews.

## Results

### Study population

Altogether, 241 visitors responded to our survey. We excluded 18 of whom payment information could not be read or ID was ambiguous, 39 who declared to have used another person´s payment card at least once and39 who did not respond to one third or more of the inquired food items. Overall, we analysed data from 145 participants. None-responders did not differ from participants regarding age (p = 0.142) and gender (p = 0.472). Altogether 84/145 (58%) participants were female. Median age was 41 years, range: 22–64 years; 80/145 (55%) stated they hold a university degree. Of 28,275 (13x15x145) possible food recalls 1,180 (0.04%) were excluded because of no response or single purchase data could not been read out from the database.

### Overall sensitivity and specificity of recall

Altogether 27,095 recalls were analysed. Of 3,523 purchased items, participants reported eating 2,268 (overall sensitivity of 72.8%), denied 846 and indecisively (Don’t know-answer) answered for 409. Of 23,572 items, actually not purchased, participants reported 20,931 as not eaten, 872 as eaten (overall specificity of 96.0%) and indecisively answered for 1,769. Altogether, participants indecisively recalled 2,178 (8.0%) food items. Median number of errors per participant was 11 with a range of 1–46. There was no significant association between the number of foods selected from the 13 investigated items and the number of reporting errors (p = 0.429). To allow better interpretation and adjustment of the results of other investigations, measures of performance of interviews are provided for each associated variable in detail in supportive information Tables A-C in S1 File.

### Influence of recall period

**Fig 1:**
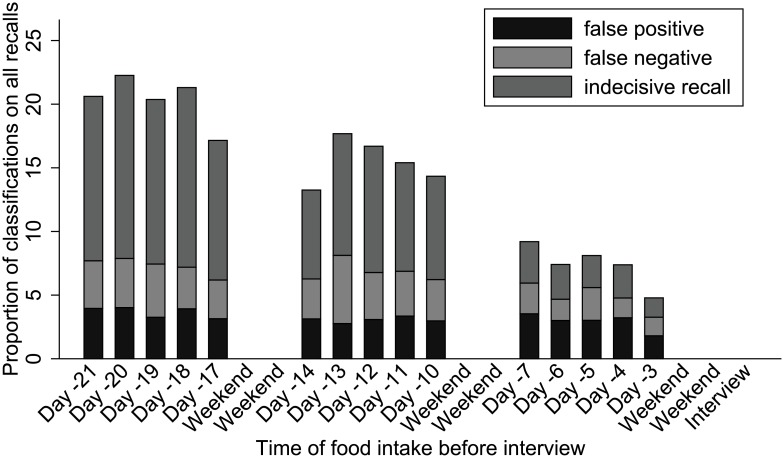
Distribution of the proportion of misclassifications of food recalls by recall period, Berlin, Germany, 2013.

All participants together paid for between 155 (day 18) and 255 (day 13) food items per day. False-negative recall increased with recall period (Fig 1). There were remarkably few bad recalls on day 14 and day 17 interrupting a continuous decline. The chance of false negative recall was twice as high after 21 days compared to 7 days (OR: 2.04; 95%-CI: 1.21, 3.45), while differences in false-positive recall are less pronounced (Table 1). In multivariable analysis, for each additional day, the chance for false-negative recall increased by 8% (OR: 1.08; 95%-CI: 1.06, 1.1), for false-positive recall by 3% (OR: 1.03; 95%-CI: 1.02, 1.05), for indecisive food recall by 12% (OR: 1.12; 95%-CI: 1.08, 1.15).

**Table 1:** Results of multivariable logistic regression of associated variables on different categories of misclassification of reported food selections, Berlin, Germany, 2013.

Associated factors	False-negative recall		False-positive recall		Indecisive recall	
(No. study participants)	Odds ratio	95%-CI	Odds ratio	95%-CI	Odds ratio	95%-CI
Recall period						
3 days	0.53	0.30, 0.95	0.50	0.31, 0.82	0.45	0.22, 0.92
4 days	0.51	0.28, 0.94	0.97	0.59, 1.57	0.81	0.60, 1.08
5 days	0.94	0.57, 1.55	0.93	0.61, 1.41	0.80	0.51, 1.24
6 days	0.53	0.30, 0.95	0.87	0.54, 1.40	0.85	0.65, 1.11
7 days	Ref	Ref	Ref	Ref	Ref	Ref
10 days	1.43	0.88, 2.32	0.84	0.50, 1.41	2.67	1.50, 4.75
11 days	1.55	0.94, 2.57	1.03	0.67, 1.60	2.89	1.59, 5.24
12 days	2.22	1.42, 3.45	0.90	0.57, 1.41	3.42	1.85, 6.33
13 days	2.29	1.43, 3.65	0.95	0.61, 1.46	3.37	1.72, 6.59
14 days	1.48	0.91, 2.41	0.98	0.63, 1.50	2.26	1.17, 4.36
17 days	1.82	1.19, 2.78	1.02	0.63, 1.65	3.80	2.04, 7.07
18 days	1.92	1.20, 3.08	1.41	0.92, 2.17	5.26	2.92, 9.46
19 days	2.35	1.47, 3.77	1.08	0.66, 1.79	4.77	2.52, 8.99
20 days	2.31	1.41, 3.78	1.38	0.87, 2.19	5.39	2.96, 9.82
21 days	2.04	1.21, 3.45	1.36	0.87, 2.11	4.75	2.53, 8.91
**Sex**						
Female (n = 84)	Ref	Ref	Ref	Ref	Ref	Ref
Male (n = 59)	0.89	0.62, 1.28	1.46	1.11, 1.91	1.79	0.86, 3.74
**Age group**						
20–29 (n = 32)	0.63	0.38, 1.06	0.77	0.52, 1.13	3.67	1.34, 10.00
30–39 (n = 36)	0.67	0.40, 1.14	0.80	0.52, 1.23	2.10	0.85, 5.15
40–49 (n = 37)	0.87	0.53, 1.43	1.14	0.82, 1.59	1.79	0.82, 3.90
50–65 (n = 37)	Ref	Ref	Ref	Ref	Ref	Ref
**University graduate**						
Yes (n = 80)	1.16	0.79, 1.69	1.02	0.77, 1.35	1.03	0.49, 2.17
No (n = 65)	Ref	Ref	Ref	Ref	Ref	Ref
**Eating vegetarian**						
Yes (n = 9)	0.77	0.40, 1.47	1.66	1.09, 2.52	4.30	1.16, 15.92
No (n = 136)	Ref	Ref	Ref	Ref	Ref	Ref
**Eating low-calorie**						
Yes (n = 10)	1.80	0.71, 4.59	1.36	0.79, 2.34	1.08	0.42, 2.76
No (n = 135)	Ref	Ref	Ref	Ref	Ref	Ref
**Food intolerance**						
Yes (n = 2)	0.31	0.14, 0.69	0.49	0.12, 2.12	22.49	4.44, 113.94
No (n = 143)	Ref	Ref	Ref	Ref	Ref	Ref
**Food item categories**						
Bakery	17.72	9.03, 34.76	0.32	0.18, 0.56	0.85	0.58, 1.23
Side dish	2.50	1.90, 3.29	1.09	0.85, 1.40	1.43	1.20, 1.70
Dessert	2.82	2.13, 3.72	1.10	0.80, 1.51	1.02	0.76, 1.35
Vegetables	2.95	2.20, 3.96	1.53	1.20, 1.95	1.25	1.06, 1.46
Main courses	Ref	Ref	Ref	Ref	Ref	Ref
Fruit Salad	32.76	10.25, 104.74	0.21	0.08, 0.51	0.76	0.55, 1.04
Salad bar	2.29	1.41, 3.71	2.23	1.49, 3.33	1.82	1.26, 2.62
Potatoes	2.74	1.65, 4.54	1.67	1.16, 2.41	1.68	1.35, 2.08
**Total (n = 145)**						

Multivariable, Odds ratio and 95% confidence interval derived from logistic regression; Recall period defined as the interval from the day of food consumption to the day of the interview in days

### Influence of type of food

Compared to the main courses, other food items were generally less accurately recalled. The use of the salad bar in the cafeteria was especially prone to false-negative recall (OR: 2.29; 95%-CI: 1.41, 3.71) as well as false-positive recall (OR: 2.23; 95%-CI: 1.49, 3.33) and indecisive recall (OR: 1.82; 95%-CI: 1.26, 2.62). Similarly, vegetables and potatoes, although less likely as food vehicles of outbreaks, were poorly recalled comparing to main courses in all three categories. False-positive recall was less likely in bakery products and fruit salad.

### Influence of demographic characteristics

The 59 males paid for 1,420 food items (24 per person) while the 84 females paid for 1,668 items (20 per person). While false-negative recall did not differ between males and females, the chance for false-positive recall was higher in males (OR 1.46; 95%-CI 1.11, 1.91). False-positive recall was also higher in vegetarians (OR 1.66; 95%-CI 1.09 2.52). False-negative recall did not vary by age or education. However, indecisive recall was more likely in vegetarians (OR: 4.30; 95%-CI: 1.16, 15.92) and in 20–29 year old participants compared to those aged 50–65 years (OR: 3.67; 95%-CI: 1.34, 10.00). Level of education of participants was not associated significantly with false-negative (OR: 1.16; 95%-CI: 0.79, 1.69), false-positive (OR: 1.02; 95%-CI: 0.77, 1.35) and indecisive recall (OR: 1.03; 95%-CI: 0.49, 2.17).

### Effort for data acquisition

Data collection based on the questionnaire required presence of 10 persons in the cafeteria for 3 hours to contact and inform visitors, receive interviewees´ informed consent, to distribute and receive the questionnaires. In comparison, to extract the data from the payment system required one staff for 2 hours.

## Discussion

This study shows that exposure misclassification can be a significant problem in the investigation of foodborne infectious disease outbreaks using data from food history interviews. The misclassification can be differential regarding the inquired food items, leading to an underestimation of measures of association of the true outbreak vehicle and false incrimination of other vehicles. For example, this scenario happened during investigation of large outbreaks of STEC in Germany caused by sprouts [7,13] and Salmonella Saintpaul in the USA caused by jalapeño and serrano peppers [14,15]. In both outbreaks epidemiological association from early studies initially identified different products. We found that the proportion of false-negative recalls is higher than false-positive, indicating that forgetting to report consumed foods is more likely than reporting food-items actually not consumed. Higher specificity and lower sensitivity of recall were reported before in a similar experiment [12].

### Influence of recall period

While false-negative recall and indecisive recall strongly increases with time, false-positive recall does not. After recall periods of two weeks or more, around 20% of all items do not get reported correctly which means lower power in epidemiological studies to detect outbreak vehicles. The high chance for false-negative recall is particularly problematic for hypothesis generation. Outbreak vehicles may be underestimated or overseen only because the exposure lies two weeks or more in the past.

### Influence of type of food

Decker et al. reported more accurate recall of more complex or distinctive dishes compared to a range of relatively similar vegetable side dishes. This is supported by our findings suggesting better recall of main courses compared to all other dishes, particularly compared to unvarying daily offerings like fruit salad and bakery. Contrarily to Decker et al., we did not find indication of significant misclassification of desserts [12]. However, better recall of main courses needs to be taken into account when evaluating explorative findings, to avoid missing vehicles in side dishes. Particularly consumption at the salad bar is poorly recalled which is in accordance with observations from an outbreak in Germany [7,8]. Unfortunately, we could not obtain information on different salad bar items as this was not included in the billing data.

### Influence of demographic characteristics

Altogether, respondent-related variables have a smaller impact than recall time and food item variables. Our findings confirm a higher chance for false-positive recall in men. This is in accordance with findings of Decker et al. [12]. Increasing age does not lead to poor recall in our study. Participants who declare being vegetarian have a higher chance for false-positive or indecisive recall despite the assumption that sensible diet leads to better recall of food consumption. However, this finding is based only on small numbers: only few study participants indicated being vegetarian (n = 9) or eating low-calorie food (n = 10).

### Effort for data acquisition

The interviews of participants required 15-times more work compared to the extraction of the electronic information from the billing system. Therefore the latter provides potential to make data collection quicker, more accurate and allows for larger study populations. However, it’s only applicable if a large proportion of cases and non-diseased persons pay cashless. An electronic interface between billing systems and databases of public health agencies might accelerate investigations.

### Limitations

Unfortunately, in our simulation study only printouts were available, demanding manual data entry. The bank as employer and the cafeteria allowed us only limited interview time. In a real scenario, such would be much longer and provide more detailed information especially regarding the different main courses and regarding individual food choices. Furthermore, the data from the payment system was only specific on the menu level and not on the choice of the visitor. Therefore, participants were not asked if they had eaten anything containing a specific ingredient and they did not have the possibility to report items which were not on the questionnaire. In a real-life scenario investigations on ingredients level might be complemented by interviews with the chefs and the kitchen staff.

One main limitation of recall-independent electronic data is that it cannot tell if paid food items were actually eaten by the participant. But we think that this misclassification is of minor importance compared with misclassification due to incorrect recall.

### Conclusion

Our results show that earliness of interviews of patients during foodborne outbreaks is essential, particularly when the pathogen and disease have long incubation periods. At least, hypothesis generating exploratory interviews should be performed before failure of recall. If available, electronic payment data for food history collection can facilitate and accelerate investigations, especially if patients are very sick or even dead. Data from our study can be used for better interpretation and adjustment of the results of surveys, case-control studies and cohort studies in outbreaks.

## Supporting information

S1 File Table A: False-negative food recalls by different groups for reported food selections, Berlin, Germany, 2013. Table caption: Univariable, Odds ratio and 95% confidence interval derived from logistic regression; CI, confidence interval; Recall period defined as the interval from the day of food consumption to the day of the interview in days. Table B: False-positive food recalls by different groups for reported food selections, Berlin, Germany, 2013. Table caption: Univariable, Odds ratio and 95% confidence interval derived from logistic regression; CI, confidence interval; Recall period defined as the interval from the day of food consumption to the day of the interview in days. Table C: Indecisive (Don’t know-answer) food recalls by different groups for reported food selections, Berlin, Germany, 2013. Table caption: Univariable, Odds ratio and 95% confidence interval derived from logistic regression; CI, confidence interval; Recall period defined as the interval from the day of food consumption to the day of the interview in days.
(DOC)

S1 Questionnaire German The questionnaire was designed using the same layout as the normal weekly menu of the cafeteria. Each column represents a working day when the canteen was open. The lines represent the 13 different food categories from which participants could choose a different serving every day. For analysis, the varying categories were grouped together: Giving 8 food item categories: main courses, side dishes, boiled potatoes (the non-varying third side dish), vegetable side dishes, desserts, fruit-salad (the non-varying third dessert), salad-bar (available every day) and bakery (available every day).
(PDF)

S1 Questionnaire English The English questionnaire is a translation of the German original. It was not used in the study but produced exclulsively to facilitate reading of this report.”
(PDF)
