# Supplementary material for: Assessment of recall error in self-reported food consumption histories among adults—Particularly delay of interviews decrease completeness of food histories—Germany, 2013
Source: PLoS One. 2017 Jun 22;12(6):e0179121. doi: 10.1371/journal.pone.0179121 (PMC5480875; doi:10.1371/journal.pone.0179121)
Supplement: S1 File — Table A: False-negative food recalls by different groups for reported food selections, Berlin, Germany, 2013. Table caption: Univariable, Odds ratio and 95% confidence interval derived from logistic regression; CI, confidence interval; Recall period defined as the interval from the day of food consumption to the day of the interview in days. Table B: False-positive food recalls by different groups for reported food selections, Berlin, Germany, 2013. Table caption: Univariable, Odds ratio and 95% confidence interval derived from logistic regression; CI, confidence interval; Recall period defined as the interval from the day of food consumption to the day of the interview in days. Table C: Indecisive (Don’t know-answer) food recalls by different groups for reported food selections, Berlin, Germany, 2013. Table caption: Univariable, Odds ratio and 95% confidence interval derived from logistic regression; CI, confidence interval; Recall period defined as the interval from the day of food consumption to the day of the interview in days. (DOC) [file pone.0179121.s001.doc]

**Supporting information table A:**

False negative food recalls by different groups for reported food selections, Berlin, Germany, 2013

| **Associated factors (n, Number of study participants)** | **Number of items paid for** | **Paid, but re-ported not eaten  (False negatives)** | **Sensitivity (%)** | **95%-CI** | **Univariable Odds ratio** | **95%-CI** |
| --- | --- | --- | --- | --- | --- | --- |
| **Recall period** |  |  |  |  |  |  |
| 3 days | 211 | 26 | 87.7 | 82.5, 91.8 | 0.53 | 0.31, 0.91 |
| 4 days | 216 | 28 | 87.0 | 81.8, 91.2 | 0.56 | 0.32, 0.99 |
| 5 days | 243 | 46 | 81.1 | 75.6, 85.8 | 0.89 | 0.56, 1.41 |
| 6 days | 226 | 30 | 86.7 | 81.6, 90.9 | 0.58 | 0.34, 0.99 |
| 7 days | 206 | 43 | 79.1 | 72.9, 84.5 | Ref | Ref |
| 10 days | 193 | 60 | 68.9 | 61.9, 75.4 | 1.71 | 1.10, 2.66 |
| 11 days | 230 | 64 | 72.2 | 65.9, 77.9 | 1.46 | 0.90, 2.38 |
| 12 days | 189 | 67 | 64.6 | 57.3, 71.4 | 2.08 | 1.37, 3.16 |
| 13 days | 255 | 97 | 62.0 | 55.7, 67.9 | 2.33 | 1.50, 3.61 |
| 14 days | 198 | 57 | 71.2 | 64.4, 77.4 | 1.53 | 0.96, 2.46 |
| 17 days | 186 | 54 | 71.0 | 63.9, 77.4 | 1.55 | 1.01, 2.37 |
| 18 days | 155 | 59 | 61.9 | 53.8, 69.6 | 2.33 | 1.50, 3.62 |
| 19 days | 215 | 77 | 64.2 | 57.4, 70.6 | 2.12 | 1.34, 3.33 |
| 20 days | 193 | 70 | 63.7 | 56.5, 70.5 | 2.16 | 1.35, 3.44 |
| 21 days | 198 | 68 | 65.7 | 58.6, 72.2 | 1.98 | 1.19, 3.29 |
| **Sex** |  |  |  |  |  |  |
| Female (n=84) | 1,668 | 477 | 71.4 | 69.2, 73.6 | Ref | Ref |
| Male (n=59) | 1,420 | 365 | 74.3 | 71.9, 76.6 | 0.86 | 0.62, 1.20 |
| **Age group** |  |  |  |  |  |  |
| 20-29 (n=32) | 714 | 160 | 77.6 | 74.4, 80.6 | 0.62 | 0.40, 0.95 |
| 30-39 (n=36) | 669 | 165 | 75.3 | 71.9, 78.6 | 0.70 | 0.44, 1.10 |
| 40-49 (n=37) | 849 | 234 | 72.4 | 69.3, 75.4 | 0.81 | 0.53, 1.25 |
| 50-65 (n=37) | 843 | 269 | 68.1 | 64.8, 71.2 | Ref | Ref |
| **University graduate** |  |  |  |  |  |  |
| Yes (n=80) | 1,788 | 490 | 72.6 | 70.5, 74.7 | 1.03 | 0.74, 1.43 |
| No (n=65) | 1,326 | 356 | 73.2 | 70.7, 75.5 | Ref | Ref |
| **Eating vegetarian** |  |  |  |  |  |  |
| Yes (n=9) | 208 | 63 | 69.7 | 63.0, 75.9 | 1.18 | 0.66, 2.09 |
| No (n=136) | 2,906 | 783 | 73.1 | 71.4, 74.7 | Ref | Ref |
| **Eating low-calorie** |  |  |  |  |  |  |
| Yes (n=10) | 212 | 91 | 57.1 | 50.1, 63.8 | 2.34 | 1.03, 4.79 |
| No (n=135) | 2,902 | 755 | 74.0 | 72.3, 75.6 | Ref | Ref |
| **Food intolerance** |  |  |  |  |  |  |
| Yes (n=2) | 24 | 2 | 91.7 | 73.0, 99.0 | 0.24 | 0.07, 0.88 |
| No (n=143) | 3,090 | 844 | 72.7 | 71.1, 74.3 | Ref | Ref |
| **Food items** |  |  |  |  |  |  |
| Bakery | 79 | 55 | 30.4 | 20.5, 41.8 | 13.51 | 7.28, 25.09 |
| Side dish | 614 | 179 | 70.8 | 67.1, 74.4 | 2.43 | 1.86, 3.17 |
| Dessert | 467 | 141 | 69.8 | 65.4, 73.9 | 2.55 | 1.93, 3.38 |
| Vegetables | 449 | 158 | 64.8 | 60.2, 69.2 | 3.20 | 2.39, 4.28 |
| Main courses | 1,062 | 154 | 85.5 | 83.2, 87.6 | Ref | Ref |
| Fruit Salad | 48 | 40 | 16.7 | 7.5, 30.2 | 29.48 | 8.73, 99.58 |
| Salad bar | 265 | 75 | 71.7 | 65.9, 77.0 | 2.33 | 1.48, 3.67 |
| Potatoes | 130 | 44 | 66.2 | 57.3, 74.2 | 3.02 | 1.89, 4.82 |
| **Total (n=145)** | **3,114** | **846** | **72.8** | **71.2, 74.4** |  |  |

Table caption: Univariable, Odds ratio and 95% confidence interval derived from logistic regression; CI, confidence interval; Recall period defined as the interval from the day of food consumption to the day of the interview in days

**Supporting information table B:**

False positive food recalls by different groups for reported food selections, Berlin, Germany, 2013

| **Associated factors**  **(n, Number of study participants)** | **Number of items not paid for** | **Reported eaten, but not paid for  (False positives)** | **Specificity (%)** | **95%-CI** | **Univariable Odds ratio** | **95%-CI** |
| --- | --- | --- | --- | --- | --- | --- |
| **Recall period** |  |  |  |  |  |  |
| 3 days | 1,538 | 32 | 97.9 | 97.1, 98.6 | 0.49 | 0.31, 0.79 |
| 4 days | 1,538 | 58 | 96.2 | 95.2, 97.1 | 0.91 | 0.57, 1.44 |
| 5 days | 1,500 | 54 | 96.4 | 95.3, 97.3 | 0.86 | 0.57, 1.29 |
| 6 days | 1,521 | 54 | 96.4 | 95.4, 97.3 | 0.85 | 0.56, 1.30 |
| 7 days | 1,518 | 63 | 95.8 | 94.7, 96.8 | Ref | Ref |
| 10 days | 1,505 | 55 | 96.3 | 95.3, 97.2 | 0.88 | 0.55, 1.39 |
| 11 days | 1,433 | 61 | 95.7 | 94.6, 96.7 | 1.03 | 0.69, 1.52 |
| 12 days | 1,446 | 56 | 96.1 | 95.0, 97.1 | 0.93 | 0.61, 1.42 |
| 13 days | 1,382 | 50 | 96.4 | 95.3, 97.3 | 0.87 | 0.56, 1.34 |
| 14 days | 1,493 | 57 | 96.2 | 95.1, 97.1 | 0.92 | 0.61, 1.37 |
| 17 days | 1,397 | 56 | 96.0 | 94.8, 97.0 | 0.96 | 0.62, 1.51 |
| 18 days | 1,397 | 71 | 94.9 | 93.6, 96.0 | 1.24 | 0.81, 1.89 |
| 19 days | 1,388 | 60 | 95.7 | 94.5, 96.7 | 1.04 | 0.64, 1.69 |
| 20 days | 1,361 | 73 | 94.6 | 93.3, 95.8 | 1.31 | 0.86, 2.00 |
| 21 days | 1,386 | 72 | 94.8 | 93.5, 95.9 | 1.27 | 0.82, 1.94 |
| **Sex** |  |  |  |  |  |  |
| Female (n=84) | 13,137 | 466 | 96.5 | 96.1, 96.8 | Ref | Ref |
| Male (n=59) | 8,382 | 401 | 95.2 | 94.7, 95.7 | 1.37 | 0.99, 1.89 |
| **Age group** |  |  |  |  |  |  |
| 20-29 (n=32) | 4,711 | 138 | 97.1 | 96.5, 97.5 | 0.69 | 0.46, 1.02 |
| 30-39 (n=36) | 5,538 | 183 | 96.7 | 96.2, 97.2 | 0.78 | 0.50, 1.21 |
| 40-49 (n=37) | 5,309 | 255 | 95.2 | 94.6, 95.8 | 1.15 | 0.78, 1.68 |
| 50-65 (n=37) | 5,767 | 243 | 95.8 | 95.2, 96.3 | Ref | Ref |
| **University graduate** |  |  |  |  |  |  |
| Yes (n=80) | 11,858 | 497 | 95.8 | 95.4, 96.2 | 1.12 | 0.79, 1.58 |
| No (n=65) | 9,945 | 375 | 96.2 | 95.8, 96.6 | Ref | Ref |
| **Eating vegetarian** |  |  |  |  |  |  |
| Yes (n=9) | 1,147 | 76 | 93.4 | 91.8, 94.7 | 1.77 | 1.12, 2.80 |
| No (n=136) | 20,656 | 796 | 96.1 | 95.9, 96.4 | Ref | Ref |
| **Eating low-calorie** |  |  |  |  |  |  |
| Yes (n=10) | 1,546 | 116 | 92.5 | 91.1, 93.8 | 2.09 | 1.12, 3.91 |
| No (n=135) | 20,257 | 756 | 96.3 | 96.0, 96.5 | Ref | Ref |
| **Food intolerance** |  |  |  |  |  |  |
| Yes (n=2) | 119 | 2 | 98.3 | 94.1, 99.8 | 0.41 | 0.10, 1.68 |
| No (n=143) | 21,684 | 870 | 96.0 | 95.7, 96.2 | Ref | Ref |
| **Food items** |  |  |  |  |  |  |
| Bakery | 1,891 | 21 | 98.9 | 98.3, 99.3 | 0.29 | 0.17, 0.52 |
| Side dish | 3,167 | 121 | 96.2 | 95.5, 96.8 | 1.04 | 0.81, 1.32 |
| Dessert | 3,417 | 134 | 96.1 | 95.4, 96.7 | 1.06 | 0.78, 1.44 |
| Vegetables | 3,356 | 183 | 94.5 | 93.7, 95.3 | 1.50 | 1.20, 1.89 |
| Main courses | 4,737 | 175 | 96.3 | 95.7, 96.8 | Ref | Ref |
| Fruit Salad | 1,923 | 23 | 98.8 | 98.2, 99.2 | 0.32 | 0.13, 0.76 |
| Salad bar | 1,583 | 116 | 92.7 | 91.3, 93.9 | 2.06 | 1.40, 3.03 |
| Potatoes | 1,729 | 99 | 94.3 | 93.1, 95.3 | 1.58 | 1.11, 2.27 |
| **Total (n=145)** | **21,803** | **872** | **96.0** | **95.7, 96.3** |  |  |

Table caption: Univariable, Odds ratio and 95% confidence interval derived from logistic regression; CI, confidence interval; Recall period defined as the interval from the day of food consumption to the day of the interview in days

**Supporting information table C:**

Indecisive (Don’t know-answer) food recalls by different groups for reported food selections, Berlin, Germany, 2013

| **Associated factors**  **(n, Number of study participants)** | **Indecisive recall (%)** | **95%-CI** | **Univariable Odds ratio** | **95%-CI** |
| --- | --- | --- | --- | --- |
| **Recall period** |  |  |  |  |
| 3 days | 1.5 | 1.0, 2.1 | 0.46 | 0.22, 0.95 |
| 4 days | 2.6 | 1.9, 3.3 | 0.80 | 0.60, 1.06 |
| 5 days | 2.5 | 1.8, 3.2 | 0.77 | 0.49, 1.21 |
| 6 days | 2.7 | 2.0, 3.5 | 0.83 | 0.63, 1.10 |
| 7 days | 3.3 | 2.4, 4.1 | Ref | Ref |
| 10 days | 8.1 | 6.9, 9.4 | 2.63 | 1.46, 4.72 |
| 11 days | 8.5 | 7.2, 9.8 | 2.77 | 1.50, 5.12 |
| 12 days | 9.9 | 8.5, 11.3 | 3.27 | 1.75, 6.14 |
| 13 days | 9.6 | 8.2, 10.9 | 3.14 | 1.60, 6.17 |
| 14 days | 7.0 | 5.8, 8.2 | 2.23 | 1.16, 4.31 |
| 17 days | 11.0 | 9.5, 12.4 | 3.66 | 1.95, 6.88 |
| 18 days | 14.1 | 12.5, 15.7 | 4.88 | 2.65, 8.99 |
| 19 days | 12.9 | 11.4, 14.5 | 4.41 | 2.30, 8.47 |
| 20 days | 14.4 | 12.8, 16.0 | 4.99 | 2.67, 9.33 |
| 21 days | 12.9 | 11.4, 14.5 | 4.41 | 2.30, 8.45 |
| **Sex** |  |  |  |  |
| Female (n=84) | 6.1 | 5.7, 6.5 | Ref | Ref |
| Male (n=59) | 11.1 | 10.6, 11.7 | 1.93 | 1.03, 3.63 |
| **Age group** |  |  |  |  |
| 20-29 (n=32) | 11.4 | 10.6, 12.2 | 2.93 | 1.22, 7.04 |
| 30-39 (n=36) | 7.5 | 6.8, 8.1 | 1.83 | 0.88, 3.83 |
| 40-49 (n=37) | 9.9 | 9.2, 10.6 | 2.49 | 1.18, 5.27 |
| 50-65 (n=37) | 4.2 | 3.7, 4.7 | Ref | Ref |
| **University graduate** |  |  |  |  |
| Yes (n=80) | 9.3 | 8.8, 9.7 | 1.44 | 0.77, 2.69 |
| No (n=65) | 6.6 | 6.2, 7.1 | Ref | Ref |
| **Eating vegetarian** |  |  |  |  |
| Yes (n=9) | 20.1 | 18.2, 22.0 | 3.20 | 0.83, 12.28 |
| No (n=136) | 7.3 | 7.0, 7.6 | Ref | Ref |
| **Eating low-calorie** |  |  |  |  |
| Yes (n=10) | 5.2 | 4.2, 6.2 | 0.60 | 0.27, 1.36 |
| No (n=135) | 8.3 | 8.0, 8.7 | Ref | Ref |
| **Food intolerance** |  |  |  |  |
| Yes (n=2) | 56.1 | 50.7, 61.5 | 15.76 | 2.47, 99.88 |
| No (n=143) | 7.5 | 7.5, 7.8 | Ref | Ref |
| **Food items** |  |  |  |  |
| Bakery | 6.0 | 5.0, 7.0 | 0.83 | 0.59, 1.16 |
| Side dish | 9.5 | 8.6, 10.3 | 1.36 | 1.15, 1.59 |
| Dessert | 7.1 | 6.4, 7.9 | 1.00 | 0.9, 1.2 |
| Vegetables | 8.5 | 7.6, 9.3 | 1.20 | 1.04, 1.39 |
| Main courses | 7.2 | 6.5, 7.8 | Ref | Ref |
| Fruit Salad | 5.5 | 4.5, 6.5 | 0.76 | 0.56, 1.02 |
| Salad bar | 11.4 | 10.0, 12.7 | 1.66 | 1.20, 2.32 |
| Potatoes | 10.7 | 9.4, 12.0 | 1.56 | 1.27, 1.91 |
| **Total (n=145)** | **8.0** | **7.7, 8.3** |  |  |

Table caption: Univariable, Odds ratio and 95% confidence interval derived from logistic regression; CI, confidence interval; Recall period defined as the interval from the day of food consumption to the day of the interview in days
