# Supplementary material for: Assessment of recall error in self-reported food consumption histories among adults—Particularly delay of interviews decrease completeness of food histories—Germany, 2013
Source: PLoS One. 2017 Jun 22;12(6):e0179121. doi: 10.1371/journal.pone.0179121 (PMC5480875; doi:10.1371/journal.pone.0179121)
Supplement: S1 Questionnaire German — Each column represents a working day when the canteen was open. The lines represent the 13 different food categories from which participants could choose a different serving every day. For analysis, the varying categories were grouped together: Giving 8 food item categories: main courses, side dishes, boiled potatoes (the non-varying third side dish), vegetable side dishes, desserts, fruit-salad (the non-varying third dessert), salad-bar (available every day) and bakery (available every day). (PDF) [file pone.0179121.s002.pdf]

ROBERT KOCH INSTITUT

Simulationsstudie Erinnerungsvermögen

FRAGEBOGEN

Fühlen Sie sich bezüglich dieser Befragung ausreichend informiert (v.a. bezüglich des Datenschutzes) und möchten Sie daran teilnehmen?

ja ☐$_1$ nein ☐$_2$

Falls ja, bitte tragen Sie hier die ID Ihrer Kantinenkarte ein:

ID Ihrer Kantinenkarte: ______________________

Falls nein, sprechen Sie gerne zur weiteren Information einen unserer Mitarbeiter an.

Geburtsjahr: _____________

Geschlecht: Frau ☐$_1$ Mann ☐$_2$

Welches ist Ihr höchster beruflicher Abschluss?

Abschluss an Universität, Hochschule ☐$_1$
Abschluss an Fachhoch/Ingenieurschule ☐$_2$
Lehre (beruflich-betriebliche Ausbildung) ☐$_3$
Berufsfach-/Handelsschule (beruflich- schulisch A.) ☐$_4$
Fachschule/Technikerschule/Fachakademie ☐$_5$
Noch in beruflicher Ausbildung ☐$_6$
Keinen Berufsabschluss ☐$_7$
Anderer Ausbildungsabschluss ☐$_8$

Haben Sie in den letzten drei Wochen hier auch Mittagessen zu sich genommen, die mit einer anderen Karte als Ihrer eigenen bezahlt wurden?

Nie ☐$_1$ Seltener als 1x die Woche ☐$_2$ Einmal die Woche oder mehr ☐$_3$ Immer ☐$_4$

Pflegen Sie eine besondere Ernährungsweise?

Nein ☐$_1$ Vegetarisch/vegan ☐$_1$ Kalorienarm ☐$_1$ Nahrungsmittelintoleranz ☐$_1$ Andere ☐$_1$

Herzlichen Dank für Ihre Teilnahme!

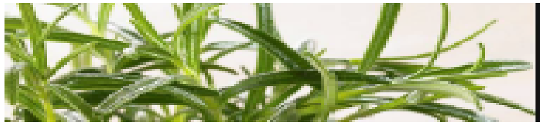

MENÜPLAN

modifiziert durch ROBERT KOCH INSTITUT

Menüplan KFW Niederlassung Berlin Charlottenstraße

Gültig vom 21.01.- 25.01.2013

	Montag 21.1.2013		Dienstag 22.1.2013		Mittwoch 23.1.2013		Donnerstag 24.1.2013		Freitag 25.1.2013	
Casino besucht ?	◯ ja ◯ nein ◯ weiß nicht		◯ ja ◯ nein ◯ weiß nicht		◯ ja ◯ nein ◯ weiß nicht		◯ ja ◯ nein ◯ weiß nicht		◯ ja ◯ nein ◯ weiß nicht	
**Haupt-komponente 1**	Kasslerkammbraten mit Nusskruste dazu Jus	◯ ja<br>◯ nein<br>◯ weiß nicht	Gratinierte Nudeln mit Putenfilet, Wirsing und Tomatensauce	◯ ja<br>◯ nein<br>◯ weiß nicht	2 Kartoffel-Pastinaken-Plätzchen auf Apfel-Rotkohl	◯ ja<br>◯ nein<br>◯ weiß nicht	**Gulasch vom Bio-Rind mit Sauerrahm**	◯ ja<br>◯ nein<br>◯ weiß nicht	**3 Bio-Schweinemedaillons im Speckmantel mit Preiselbeersauce**	◯ ja<br>◯ nein<br>◯ weiß nicht
**Haupt-komponente 2**	„Rabas Empanadas „– Panierte Tintenfischstreifen dazu Chili-Pfeffer-Mayonaise	◯ ja<br>◯ nein<br>◯ weiß nicht	5 Balkanröllchen in Paprikasauce	◯ ja<br>◯ nein<br>◯ weiß nicht	Hähnchengeschnetzeltes Power and Sweet mit Wokgemüse	◯ ja<br>◯ nein<br>◯ weiß nicht	Ribolitta – Eintopfgericht mit Hühnchen, Kohl und Dicken Bohnen	◯ ja<br>◯ nein<br>◯ weiß nicht	Wildlachsschnitte mit Orangensauce	◯ ja<br>◯ nein<br>◯ weiß nicht
**Haupt-komponente 3**	Rigatoni mit Gemüse-Bolognese	◯ ja<br>◯ nein<br>◯ weiß nicht	Zucchini gefüllt mit Gemüse an Kräutersauce dazu Graupenrisotto	◯ ja<br>◯ nein<br>◯ weiß nicht	Kohlroulade mit Hackfleischfüllung und Kümmel-Speck-Sauce	◯ ja<br>◯ nein<br>◯ weiß nicht	2 Semmelknödel mit Schwammerlsauce (Mischpilze)	◯ ja<br>◯ nein<br>◯ weiß nicht	Käsespätzle „Allgäuer Art“	◯ ja<br>◯ nein<br>◯ weiß nicht
**Gemüse 1**	Frisches Lauchgemüse	◯ ja<br>◯ nein<br>◯ weiß nicht	Maisgemüse	◯ ja<br>◯ nein<br>◯ weiß nicht	Wokgemüse	◯ ja<br>◯ nein<br>◯ weiß nicht	**Bio-Wirsing**	◯ ja<br>◯ nein<br>◯ weiß nicht	**Bio-Champignongemüse**	◯ ja<br>◯ nein<br>◯ weiß nicht
**Gemüse 2**	**Tomatengemüse**	◯ ja<br>◯ nein<br>◯ weiß nicht	Okragemüse mit Tomaten	◯ ja<br>◯ nein<br>◯ weiß nicht	Apfelrotkraut	◯ ja<br>◯ nein<br>◯ weiß nicht	Buttererbsen	◯ ja<br>◯ nein<br>◯ weiß nicht	Apfel-Sauerkraut	◯ ja<br>◯ nein<br>◯ weiß nicht
**Beilage 1**	**Schupfnudeln**	◯ ja<br>◯ nein<br>◯ weiß nicht	Graupenrisotto	◯ ja<br>◯ nein<br>◯ weiß nicht	Kartoffelschnee	◯ ja<br>◯ nein<br>◯ weiß nicht	**Bio-Bandnudeln**	◯ ja<br>◯ nein<br>◯ weiß nicht	**Möhren**-Ingwer-Stampf	◯ ja<br>◯ nein<br>◯ weiß nicht
**Beilage 2**	Kartoffel-Olivenpüree	◯ ja<br>◯ nein<br>◯ weiß nicht	1 Maispuffer	◯ ja<br>◯ nein<br>◯ weiß nicht	Mie-Nudeln	◯ ja<br>◯ nein<br>◯ weiß nicht	1 Semmelknödel	◯ ja<br>◯ nein<br>◯ weiß nicht	**Bio-Langkornreis**	◯ ja<br>◯ nein<br>◯ weiß nicht
**Salzkartoffeln (tgl. Beilage 3)**	Salzkartoffeln	◯ ja<br>◯ nein<br>◯ weiß nicht	Salzkartoffeln	◯ ja<br>◯ nein<br>◯ weiß nicht	Salzkartoffeln	◯ ja<br>◯ nein<br>◯ weiß nicht	Salzkartoffeln	◯ ja<br>◯ nein<br>◯ weiß nicht	Salzkartoffeln	◯ ja<br>◯ nein<br>◯ weiß nicht
**Salatbar (tgl. angeboten)**	Rohkost, Mariniertes o.a. Produkte der Salatbar?	◯ ja<br>◯ nein<br>◯ weiß nicht	Rohkost, Mariniertes o.a. Produkte der Salatbar?	◯ ja<br>◯ nein<br>◯ weiß nicht	Rohkost, Mariniertes o.a. Produkte der Salatbar?	◯ ja<br>◯ nein<br>◯ weiß nicht	Rohkost, Mariniertes o.a. Produkte der Salatbar?	◯ ja<br>◯ nein<br>◯ weiß nicht	Rohkost, Mariniertes o.a. Produkte der Salatbar?	◯ ja<br>◯ nein<br>◯ weiß nicht
**Dessert 1**	Quark-Mousse mit Apfelkompott	◯ ja<br>◯ nein<br>◯ weiß nicht	Haselnusspudding	◯ ja<br>◯ nein<br>◯ weiß nicht	Eierpfannkuchen mit Rosine-Quark, Vanillesauce (warmes Dessert)	◯ ja<br>◯ nein<br>◯ weiß nicht	Vanillecreme mit Blaubeerpüree	◯ ja<br>◯ nein<br>◯ weiß nicht	Schwarzwälder Schokomus	◯ ja<br>◯ nein<br>◯ weiß nicht
**Dessert 2**	Fruchtjoghurt	◯ ja<br>◯ nein<br>◯ weiß nicht	Bananenquark	◯ ja<br>◯ nein<br>◯ weiß nicht	Mousse au Chocolat	◯ ja<br>◯ nein<br>◯ weiß nicht	Madarinenjoghurt	◯ ja<br>◯ nein<br>◯ weiß nicht	Apfel-Rosinenquark	◯ ja<br>◯ nein<br>◯ weiß nicht
**Obstsalat (tgl. Dessert 3)**	Obstsalat	◯ ja<br>◯ nein<br>◯ weiß nicht	Obstsalat	◯ ja<br>◯ nein<br>◯ weiß nicht	Obstsalat	◯ ja<br>◯ nein<br>◯ weiß nicht	Obstsalat	◯ ja<br>◯ nein<br>◯ weiß nicht	Obstsalat	◯ ja<br>◯ nein<br>◯ weiß nicht
**Backwaren (tgl. angeboten)**	Brötchen oder Scheibe Brot, pur	◯ ja<br>◯ nein<br>◯ weiß nicht	Brötchen oder Scheibe Brot, pur	◯ ja<br>◯ nein<br>◯ weiß nicht	Brötchen oder Scheibe Brot, pur	◯ ja<br>◯ nein<br>◯ weiß nicht	Brötchen oder Scheibe Brot, pur	◯ ja<br>◯ nein<br>◯ weiß nicht	Brötchen oder Scheibe Brot, pur	◯ ja<br>◯ nein<br>◯ weiß nicht

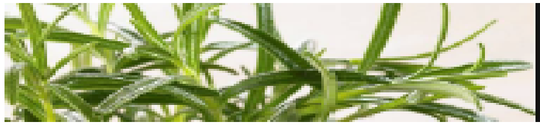

MENÜPLAN

modifiziert durch ROBERT KOCH INSTITUT

Menüplan KFW Niederlassung Berlin Charlottenstraße

Gültig vom 14.01.- 18.01.2013

	**Montag 14.1.2013**		**Dienstag 15.1.2013**		**Mittwoch 16.1.2013**		**Donnerstag 17.1.2013**		**Freitag 18.1.2013**	
Casino besucht ?	◯ ja ◯ nein ◯ weiß nicht		◯ ja ◯ nein ◯ weiß nicht		◯ ja ◯ nein ◯ weiß nicht		◯ ja ◯ nein ◯ weiß nicht		◯ ja ◯ nein ◯ weiß nicht	
**Haupt-komponente 1**	Veltener Schweinenackensteak mit Kräuterbutter oder Jus	◯ ja ◯ nein ◯ weiß nicht	**Bio-Edamer Käseschnitzel mit verschiedenen Dips**	◯ ja ◯ nein ◯ weiß nicht	**Bio-Paprikaschote mit Hackfleischfüllung und Tomatensauce**	◯ ja ◯ nein ◯ weiß nicht	**Bio-Züricher Kalbsgeschnetzeltes mit Champignons**	◯ ja ◯ nein ◯ weiß nicht	Gebratener Leberkäse mit Sauce oder süßem Senf	◯ ja ◯ nein ◯ weiß nicht
**Haupt-komponente 2**	Gemüsereispfanne mit Putenhackbällchen	◯ ja ◯ nein ◯ weiß nicht	Forelle Müllerin Art mit zerlassener Butter	◯ ja ◯ nein ◯ weiß nicht	Gulasch vom Rind mit Sauerrahm	◯ ja ◯ nein ◯ weiß nicht	Gebratene Hähnchenbrust mit Honig-Pfeffersauce	◯ ja ◯ nein ◯ weiß nicht	Raviolini Formaggio mit Tomaten-Basilikum-Sauce dazu geriebener Parmesan	◯ ja ◯ nein ◯ weiß nicht
**Haupt-komponente 3**	Frühlingsrolle mit Gemüsefüllung dazu Sweet-Chilli-Sauce	◯ ja ◯ nein ◯ weiß nicht	Dönerfleisch vom Hähnchen mit Joghurtsauce	◯ ja ◯ nein ◯ weiß nicht	Mangold-Möhren-Rösti mit Frischkäse-Tomaten-Dip	◯ ja ◯ nein ◯ weiß nicht	Blumenkohl-Kartoffelauflauf mit Soja-Bolognese	◯ ja ◯ nein ◯ weiß nicht	Pochierte Fischroulade auf Gemüsestreifen dazu Basilikumsauce	◯ ja ◯ nein ◯ weiß nicht
**Gemüse 1**	Pilzegemüse	◯ ja ◯ nein ◯ weiß nicht	Geschmolzene Kirschtomaten	◯ ja ◯ nein ◯ weiß nicht	Hausgemachter Rotkohl	◯ ja ◯ nein ◯ weiß nicht	**Bio-Fenchel**	◯ ja ◯ nein ◯ weiß nicht	Chicorée gedünstet	◯ ja ◯ nein ◯ weiß nicht
**Gemüse 2**	**Frische Steckrüben**	◯ ja ◯ nein ◯ weiß nicht	Mais	◯ ja ◯ nein ◯ weiß nicht	Leipziger Gemüse	◯ ja ◯ nein ◯ weiß nicht	Frische Möhren	◯ ja ◯ nein ◯ weiß nicht	Blumenkohl	◯ ja ◯ nein ◯ weiß nicht
**Beilage 1**	**Lauwarmer Linsensalat**	◯ ja ◯ nein ◯ weiß nicht	Reisnudeln	◯ ja ◯ nein ◯ weiß nicht	Bio-Langkornreis	◯ ja ◯ nein ◯ weiß nicht	**Bio-Bandnudeln**	◯ ja ◯ nein ◯ weiß nicht	Kartoffelpüree	◯ ja ◯ nein ◯ weiß nicht
**Beilage 2**	Bratkartoffeln mit Zwiebeln	◯ ja ◯ nein ◯ weiß nicht	Gebackene Kartoffeln	◯ ja ◯ nein ◯ weiß nicht	Semmelknödel	◯ ja ◯ nein ◯ weiß nicht	Günkernrisotto	◯ ja ◯ nein ◯ weiß nicht	Gemischter Wildreis	◯ ja ◯ nein ◯ weiß nicht
**Salzkartoffeln (tgl. Beilage 3)**	Salzkartoffeln	◯ ja ◯ nein ◯ weiß nicht	Salzkartoffeln	◯ ja ◯ nein ◯ weiß nicht	Salzkartoffeln	◯ ja ◯ nein ◯ weiß nicht	Salzkartoffeln	◯ ja ◯ nein ◯ weiß nicht	Salzkartoffeln	◯ ja ◯ nein ◯ weiß nicht
**Salatbar (tgl. angeboten)**	Rohkost, Mariniertes o.a. Produkte der Salatbar?	◯ ja ◯ nein ◯ weiß nicht	Rohkost, Mariniertes o.a. Produkte der Salatbar?	◯ ja ◯ nein ◯ weiß nicht	Rohkost, Mariniertes o.a. Produkte der Salatbar?	◯ ja ◯ nein ◯ weiß nicht	Rohkost, Mariniertes o.a. Produkte der Salatbar?	◯ ja ◯ nein ◯ weiß nicht	Rohkost, Mariniertes o.a. Produkte der Salatbar?	◯ ja ◯ nein ◯ weiß nicht
**Dessert 1**	Panna Cotta mit Beerensauce	◯ ja ◯ nein ◯ weiß nicht	Cappuccinomousse	◯ ja ◯ nein ◯ weiß nicht	Zitronenquark	◯ ja ◯ nein ◯ weiß nicht	Weißweinbirne mit Schokoladen-Maronencreme	◯ ja ◯ nein ◯ weiß nicht	Dampfnudel mit Kruste dazu Vanillesauce	◯ ja ◯ nein ◯ weiß nicht
**Dessert 2**	Buttermilch-Vanillecreme mit Mandarinen	◯ ja ◯ nein ◯ weiß nicht	Kokosmousse	◯ ja ◯ nein ◯ weiß nicht	Bananen-Ananas-Götterspeise mit Sahne	◯ ja ◯ nein ◯ weiß nicht	Früchtejoghurt	◯ ja ◯ nein ◯ weiß nicht	Vanillepudding mit Schokosauce	◯ ja ◯ nein ◯ weiß nicht
**Obstsalat (tgl. Dessert 3)**	Obstsalat	◯ ja ◯ nein ◯ weiß nicht	Obstsalat	◯ ja ◯ nein ◯ weiß nicht	Obstsalat	◯ ja ◯ nein ◯ weiß nicht	Obstsalat	◯ ja ◯ nein ◯ weiß nicht	Obstsalat	◯ ja ◯ nein ◯ weiß nicht
**Backwaren (tgl. angeboten)**	Brötchen oder Scheibe Brot, pur	◯ ja ◯ nein ◯ weiß nicht	Brötchen oder Scheibe Brot, pur	◯ ja ◯ nein ◯ weiß nicht	Brötchen oder Scheibe Brot, pur	◯ ja ◯ nein ◯ weiß nicht	Brötchen oder Scheibe Brot, pur	◯ ja ◯ nein ◯ weiß nicht	Brötchen oder Scheibe Brot, pur	◯ ja ◯ nein ◯ weiß nicht

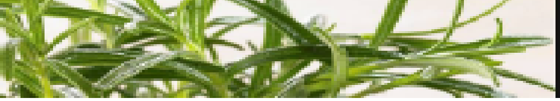

MENÜPLAN

modifiziert durch ROBERT KOCH INSTITUT

Menüplan KFW Niederlassung Berlin Charlottenstraße

Gültig vom 07.01.- 11.01.2013

	Montag 7.1.13		Dienstag 8.1.13		Mittwoch 9.1.13		Donnerstag 10.1.13		Freitag 11.1.13	
**Casino besucht ?**	◯ ja ◯ nein ◯ weiß nicht		◯ ja ◯ nein ◯ weiß nicht		◯ ja ◯ nein ◯ weiß nicht		◯ ja ◯ nein ◯ weiß nicht		◯ ja ◯ nein ◯ weiß nicht	
**Haupt-komponente 1**	Putenkeulenspieß mit Sweet-Chilli-Sauce	◯ ja<br>◯ nein<br>◯ weiß nicht	**Bio-Eieromlette mit Champignonfüllung dazu Kräuterdip**	◯ ja<br>◯ nein<br>◯ weiß nicht	Brandenburger Wurstgulasch	◯ ja<br>◯ nein<br>◯ weiß nicht	Rindergeschnetzeltes Stroganoff	◯ ja<br>◯ nein<br>◯ weiß nicht	**Bio-Currywurst (100g) mit Gewürzketchup**	◯ ja<br>◯ nein<br>◯ weiß nicht
**Haupt-komponente 2**	Bratwurstschnecke mit Sauce oder süßem Senf	◯ ja<br>◯ nein<br>◯ weiß nicht	Paniertes Seelachsfilet mit Remouladensauce	◯ ja<br>◯ nein<br>◯ weiß nicht	Lammhüfte gebraten dazu Sauce von roten Zwiebeln	◯ ja<br>◯ nein<br>◯ weiß nicht	Barbarieentenbrust gebraten mit Balsamico-Honigsauce	◯ ja<br>◯ nein<br>◯ weiß nicht	Welsfilet in Senfmarinade dazu Dillsauce	◯ ja<br>◯ nein<br>◯ weiß nicht
**Haupt-komponente 3**	2 Kartoffeltaschen gefüllt mit Tomaten und Mozarella	◯ ja<br>◯ nein<br>◯ weiß nicht	Gebratene Hähnchenbrust mit Käsesauce	◯ ja<br>◯ nein<br>◯ weiß nicht	Linsenfrikadelle auf Paprika-Pilzragout	◯ ja<br>◯ nein<br>◯ weiß nicht	Gnocchi mit gehackten Wallnusskernen ,Trüffelöl u. Weißweinsauce	◯ ja<br>◯ nein<br>◯ weiß nicht	Raviolini mit Gemüse-füllung in Gorgonzola -sauce mit Tomaten	◯ ja<br>◯ nein<br>◯ weiß nicht
**Gemüse 1**	Weinsauerkraut	◯ ja<br>◯ nein<br>◯ weiß nicht	Frische Möhren	◯ ja<br>◯ nein<br>◯ weiß nicht	Bohnengemüse	◯ ja<br>◯ nein<br>◯ weiß nicht	Frisches Kürbis-Karottengemüse	◯ ja<br>◯ nein<br>◯ weiß nicht	Fenchel	◯ ja<br>◯ nein<br>◯ weiß nicht
**Gemüse 2**	Brokkoli	◯ ja<br>◯ nein<br>◯ weiß nicht	**Bio-Rosenkohl**	◯ ja<br>◯ nein<br>◯ weiß nicht	Paprika-Pilzragout	◯ ja<br>◯ nein<br>◯ weiß nicht	Grünkohl	◯ ja<br>◯ nein<br>◯ weiß nicht	Rahmwirsing	◯ ja<br>◯ nein<br>◯ weiß nicht
**Beilage 1**	**Bio-Spätzle**	◯ ja<br>◯ nein<br>◯ weiß nicht	Basmatireis	◯ ja<br>◯ nein<br>◯ weiß nicht	Spiralnudeln	◯ ja<br>◯ nein<br>◯ weiß nicht	4 Macairkartoffeln	◯ ja<br>◯ nein<br>◯ weiß nicht	Hausgemachte Bio-Kartoffelwedges	◯ ja<br>◯ nein<br>◯ weiß nicht
**Beilage 1**	Bratkartoffeln	◯ ja<br>◯ nein<br>◯ weiß nicht	Lauwarmer Kartoffelsalat	◯ ja<br>◯ nein<br>◯ weiß nicht	Rosmarinkartoffeln	◯ ja<br>◯ nein<br>◯ weiß nicht	Polentapüree	◯ ja<br>◯ nein<br>◯ weiß nicht	Butterreis	◯ ja<br>◯ nein<br>◯ weiß nicht
**Salzkartoffeln (tgl. Beilage 3)**	Salzkartoffeln	◯ ja<br>◯ nein<br>◯ weiß nicht	Salzkartoffeln	◯ ja<br>◯ nein<br>◯ weiß nicht	Salzkartoffeln	◯ ja<br>◯ nein<br>◯ weiß nicht	Salzkartoffeln	◯ ja<br>◯ nein<br>◯ weiß nicht	Salzkartoffeln	◯ ja<br>◯ nein<br>◯ weiß nicht
**Salatbar**	Rohkost, Mariniertes o.a. Produkte der Salatbar?	◯ ja<br>◯ nein<br>◯ weiß nicht	Rohkost, Mariniertes o.a. Produkte der Salatbar?	◯ ja<br>◯ nein<br>◯ weiß nicht	Rohkost, Mariniertes o.a. Produkte der Salatbar?	◯ ja<br>◯ nein<br>◯ weiß nicht	Rohkost, Mariniertes o.a. Produkte der Salatbar?	◯ ja<br>◯ nein<br>◯ weiß nicht	Rohkost, Mariniertes o.a. Produkte der Salatbar?	◯ ja<br>◯ nein<br>◯ weiß nicht
**Dessert 1**	Grießpudding mit Apfelmus	◯ ja<br>◯ nein<br>◯ weiß nicht	Marzipanmousse	◯ ja<br>◯ nein<br>◯ weiß nicht	Panna Cotta mit Anispflaumen	◯ ja<br>◯ nein<br>◯ weiß nicht	Apfelstrudel mit Vanillesauce	◯ ja<br>◯ nein<br>◯ weiß nicht	Haselnusspudding	◯ ja<br>◯ nein<br>◯ weiß nicht
**Dessert 2**	Früchtecreme	◯ ja<br>◯ nein<br>◯ weiß nicht	Buttermilchdessert mit Kirschen	◯ ja<br>◯ nein<br>◯ weiß nicht	Cappuchino-Creme	◯ ja<br>◯ nein<br>◯ weiß nicht	Mousse au chocolat	◯ ja<br>◯ nein<br>◯ weiß nicht	Früchtejoghurt	◯ ja<br>◯ nein<br>◯ weiß nicht
**Obstsalat (tgl. Dessert 3)**	Obstsalat	◯ ja<br>◯ nein<br>◯ weiß nicht	Obstsalat	◯ ja<br>◯ nein<br>◯ weiß nicht	Obstsalat	◯ ja<br>◯ nein<br>◯ weiß nicht	Obstsalat	◯ ja<br>◯ nein<br>◯ weiß nicht	Obstsalat	◯ ja<br>◯ nein<br>◯ weiß nicht
**Backwaren**	Brötchen oder Scheibe Brot,	◯ ja<br>◯ nein<br>◯ weiß nicht	Brötchen oder Scheibe Brot,	◯ ja<br>◯ nein<br>◯ weiß nicht	Brötchen oder Scheibe Brot,	◯ ja<br>◯ nein<br>◯ weiß nicht	Brötchen oder Scheibe Brot,	◯ ja<br>◯ nein<br>◯ weiß nicht	Brötchen oder Scheibe Brot,	◯ ja<br>◯ nein<br>◯ weiß nicht
