# Supplementary material for: Assessment of recall error in self-reported food consumption histories among adults—Particularly delay of interviews decrease completeness of food histories—Germany, 2013
Source: PLoS One. 2017 Jun 22;12(6):e0179121. doi: 10.1371/journal.pone.0179121 (PMC5480875; doi:10.1371/journal.pone.0179121)
Supplement: S1 Questionnaire English — It was not used in the study but produced exclulsively to facilitate reading of this report.” (PDF) [file pone.0179121.s003.pdf]

ROBERT KOCH INSTITUT

# Simulation Study

## Questionnaire

Do you feel sufficiently informed (particularly with regards to data safety) and do you like to participate?

Yes ◯ No ◯

If yes, please provide here the ID-number of your canteen visitor card/badge:________________

If not, please ask any member of our research team in the canteen.

Year of birth:___________________

Gender/sex: female ◯ male ◯

Please choose and tick your highest level of education from the list below:

University degree ◯

Polytechnic degree ◯

Professional training qualification ◯

Business school qualification ◯

Private academy ◯

No professional training qualificationg ◯

Did you ever have lunch in the canteen which was paid with another person´s canteen-visitor-card (during the last 3 weeks)?

Never ◯ less than once a week ◯ once a week or more ◯ always ◯

Do you practice a certain diet?

No ◯ vegan/vegetarian ◯ low-calorie ◯ any intolerance ◯ any other ◯

Thank you very much for your participation!

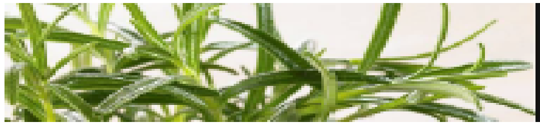

# MENÜPLAN

Modified by ROBERT KOCH INSTITUT

Menüplan KFW Niederlassung Berlin Charlottenstraße

**valid from 21.01.- 25.01.2013**

| | Monday 21.1.2013 | | Tuesday 22.1.2013 | | Wednesday 23.1.2013 | | Thursday 24.1.2013 | | Friday 25.1.2013 | |
|---|---|---|---|---|---|---|---|---|---|---|
| I visited the canteen? | ◯ Yes ◯ No ◯ do not remember | | ◯ Yes ◯ No ◯ do not remember | | ◯ Yes ◯ No ◯ do not remember | | ◯ Yes ◯ No ◯ do not remember | | ◯ Yes ◯No ◯ do not remember | |
| **Maincourse 1** | Smoked por roast with nut crust | ◯ Yes<br>◯ No<br>◯ no recall | Gratinierte Nudeln mit Putenfilet, Wirsing und Tomatensauce | ◯ Yes<br>◯ No<br>◯ no recall | 2 Kartoffel-Pastinaken-Plätzchen auf Apfel-Rotkohl | ◯ Yes<br>◯ No<br>◯ no recall | **Gulasch vom Bio-Rind mit Sauerrahm** | ◯ Yes<br>◯ No<br>◯ no recall | **3 Bio-Schweinemedaillons im Speckmantel mit Preiselbeersauce** | ◯ Yes<br>◯ No<br>◯ no recall |
| **Maincourse 2** | „Rabas Empanadas „– Breaded calamari with Chili-Pepper-Mayonaise | ◯ Yes<br>◯ No<br>◯ no recall | 5 Balkanröllchen in Paprikasauce | ◯ Yes<br>◯ No<br>◯ no recall | Hähnchengeschnetzeltes Power and Sweet mit Wokgemüse | ◯ Yes<br>◯ No<br>◯ no recall | Ribolitta – Eintopfgericht mit Hühnchen, Kohl und Dicken Bohnen | ◯ Yes<br>◯ No<br>◯ no recall | Wildlachsschnitte mit Orangensauce | ◯ Yes<br>◯ No<br>◯ no recall |
| **Maincourse 3** | Rigatoni vegetable-bolognese | ◯ Yes<br>◯ No<br>◯ no recall | Zucchini gefüllt mit Gemüse an Kräutersauce dazu Graupenrisotto | ◯ Yes<br>◯ No<br>◯ no recall | Kohlroulade mit Hackfleischfüllung und Kümmel-Speck-Sauce | ◯ Yes<br>◯ No<br>◯ no recall | 2 Semmelknödel mit Schwammerlsauce (Mischpilze) | ◯ Yes<br>◯ No<br>◯ no recall | Käsespätzle „Allgäuer Art" | ◯ Yes<br>◯ No<br>◯ no recall |
| **Vegetable side dish 1** | Fresh garden leek | ◯ Yes<br>◯ No<br>◯ no recall | Maisgemüse | ◯ Yes<br>◯ No<br>◯ no recall | Wokgemüse | ◯ Yes<br>◯ No<br>◯ no recall | **Bio-Wirsing** | ◯ Yes<br>◯ No<br>◯ no recall | **Bio-Champignongemüse** | ◯ Yes<br>◯ No<br>◯ no recall |
| **Vegetable Side dish 2** | **Tomatoes** | ◯ Yes<br>◯ No<br>◯ no recall | Okragemüse mit Tomaten | ◯ Yes<br>◯ No<br>◯ no recall | Apfelrotkraut | ◯ Yes<br>◯ No<br>◯ no recall | Buttererbsen | ◯ Yes<br>◯ No<br>◯ no recall | Apfel-Sauerkraut | ◯ Yes<br>◯ No<br>◯ no recall |
| **Side dish 1** | **Bavarian Potatoe noodles** | ◯ Yes<br>◯ No<br>◯ no recall | Graupenrisotto | ◯ Yes<br>◯ No<br>◯ no recall | Kartoffelschnee | ◯ Yes<br>◯ No<br>◯ no recall | **Bio-Bandnudeln** | ◯ Yes<br>◯ No<br>◯ no recall | **Möhren**-Ingwer-Stampf | ◯ Yes<br>◯ No<br>◯ no recall |
| **Side dish 2** | Mashed potatoes with olives | ◯ Yes<br>◯ No<br>◯ no recall | 1 Maispuffer | ◯ Yes<br>◯ No<br>◯ no recall | Mie-Nudeln | ◯ Yes<br>◯ No<br>◯ no recall | 1 Semmelknödel | ◯ Yes<br>◯ No<br>◯ no recall | **Bio-Langkornreis** | ◯ Yes<br>◯ No<br>◯ no recall |
| **Boiled potatoes (daily served side dish 3)** | Boiled potatoes | ◯ Yes<br>◯ No<br>◯ no recall | Boiled potatoes | ◯ Yes<br>◯ No<br>◯ no recall | Boiled potatoes | ◯ Yes<br>◯ No<br>◯ no recall | Boiled potatoes | ◯ Yes<br>◯ No<br>◯ no recall | Boiled potatoes | ◯ Yes<br>◯ No<br>◯ no recall |
| **Salad bar (available evry day)** | Any item from the salad bar | ◯ Yes<br>◯ No<br>◯ no recall | Any item from the salad bar | ◯ Yes<br>◯ No<br>◯ no recall | Any item from the salad bar | ◯ Yes<br>◯ No<br>◯ no recall | Rohkost, Mariniertes o.a. Produkte der Salatbar? | ◯ Yes<br>◯ No<br>◯ no recall | Rohkost, Mariniertes o.a. Produkte der Salatbar? | ◯ Yes<br>◯ No<br>◯ no recall |
| **Dessert 1** | Quark-Mousse mit Apfelkompott | ◯ Yes<br>◯ No<br>◯ no recall | Haselnusspudding | ◯ Yes<br>◯ No<br>◯ no recall | Eierpfannkuchen mit Rosine-Quark, Vanillesauce (warmes Dessert) | ◯ Yes<br>◯ No<br>◯ no recall | Vanillecreme mit Blaubeerpüree | ◯ Yes<br>◯ No<br>◯ no recall | Schwarzwälder Schokomus | ◯ Yes<br>◯ No<br>◯ no recall |
| **Dessert 2** | Fruchtjoghurt | ◯ Yes<br>◯ No<br>◯ no recall | Bananenquark | ◯ Yes<br>◯ No<br>◯ no recall | Mousse au Chocolat | ◯ Yes<br>◯ No<br>◯ no recall | Madarinenjoghurt | ◯ Yes<br>◯ No<br>◯ no recall | Apfel-Rosinenquark | ◯ Yes<br>◯ No<br>◯ no recall |
| **Fruits salad (daily served dessert 3)** | Fruit salad | ◯ Yes<br>◯ No<br>◯ no recall | Fruit salad | ◯ Yes<br>◯ No<br>◯ no recall | Fruit salad | ◯ Yes<br>◯ No<br>◯ no recall | Fruit salad | ◯ Yes<br>◯ No<br>◯ no recall | Fruit salad | ◯ Yes<br>◯ No<br>◯ no recall |
| **Backwaren** [illegible] | Roll or slice d bread | ◯ Yes | Roll or slice d bread | ◯ Yes | Roll or slice d bread | ◯ Yes | Roll or slice d bread | ◯ Yes | Roll or slice d bread | ◯ Yes |

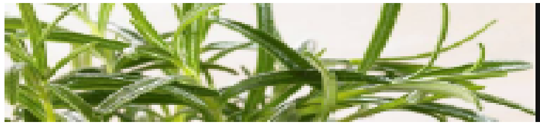

# MENÜPLAN

Modified by ROBERT KOCH INSTITUT

Menüplan KFW Niederlassung Berlin Charlottenstraße

**valid from 14.01.- 18.01.2013**

| | **Monday 14.1.2013** | **Tuesday 15.1.2013** | **Wednesday 16.1.2013** | **Thursday 17.1.2013** | **Friday 18.1.2013** |
|---|---|---|---|---|---|
| I visited the canteen? | ◯ Yes ◯ No ◯ do not remember | ◯ Yes ◯ No ◯ do not remember | ◯ Yes ◯ No ◯ do not remember | ◯ Yes ◯ No ◯ do not remember | ◯ Yes ◯No ◯ do not remember |
| **Maincourse 1** | Veltener Schweinenackensteak mit Kräuterbutter oder Jus ◯ Yes ◯ No ◯ no recall | **Bio-Edamer Käseschnitzel mit verschiedenen Dips** ◯ Yes ◯ No ◯ no recall | **Bio-Paprikaschote mit Hackfleischfüllung und Tomatensauce** ◯ Yes ◯ No ◯ no recall | **Bio-Züricher Kalbsgeschnetzeltes mit Champignons** ◯ Yes ◯ No ◯ no recall | Gebratener Leberkäse mit Sauce oder süßem Senf ◯ Yes ◯ No ◯ no recall |
| **Maincourse 2** | Gemüsereispfanne mit Putenhackbällchen ◯ Yes ◯ No ◯ no recall | Forelle Müllerin Art mit zerlassener Butter ◯ Yes ◯ No ◯ no recall | Gulasch vom Rind mit Sauerrahm ◯ Yes ◯ No ◯ no recall | Gebratene Hähnchenbrust mit Honig-Pfeffersauce ◯ Yes ◯ No ◯ no recall | Raviolini Formaggio mit Tomaten-Basilikum-Sauce dazu geriebener Parmesan ◯ Yes ◯ No ◯ no recall |
| **Maincourse 3** | Frühlingsrolle mit Gemüsefüllung dazu Sweet-Chilli-Sauce ◯ Yes ◯ No ◯ no recall | Dönerfleisch vom Hähnchen mit Joghurtsauce ◯ Yes ◯ No ◯ no recall | Mangold-Möhren-Rösti mit Frischkäse-Tomaten-Dip ◯ Yes ◯ No ◯ no recall | Blumenkohl-Kartoffelauflauf mit Soja-Bolognese ◯ Yes ◯ No ◯ no recall | Pochierte Fischroulade auf Gemüsestreifen dazu Basilikumsauce ◯ Yes ◯ No ◯ no recall |
| **Vegetable side dish 1** | Pilzegemüse ◯ Yes ◯ No ◯ no recall | Geschmolzene Kirschtomaten ◯ Yes ◯ No ◯ no recall | Hausgemachter Rotkohl ◯ Yes ◯ No ◯ no recall | **Bio-Fenchel** ◯ Yes ◯ No ◯ no recall | Chicorée gedünstet ◯ Yes ◯ No ◯ no recall |
| **Vegetable Side dish 2** | **Frische Steckrüben** ◯ Yes ◯ No ◯ no recall | Mais ◯ Yes ◯ No ◯ no recall | Leipziger Gemüse ◯ Yes ◯ No ◯ no recall | Frische Möhren ◯ Yes ◯ No ◯ no recall | Blumenkohl ◯ Yes ◯ No ◯ no recall |
| **Side dish 1** | **Lauwarmer Linsensalat** ◯ Yes ◯ No ◯ no recall | Reisnudeln ◯ Yes ◯ No ◯ no recall | Bio-Langkornreis ◯ Yes ◯ No ◯ no recall | **Bio-Bandnudeln** ◯ Yes ◯ No ◯ no recall | Kartoffelpüree ◯ Yes ◯ No ◯ no recall |
| **Side dish 2** | Bratkartoffeln mit Zwiebeln ◯ Yes ◯ No ◯ no recall | Gebackene Kartoffeln ◯ Yes ◯ No ◯ no recall | Semmelknödel ◯ Yes ◯ No ◯ no recall | Günkernrisotto ◯ Yes ◯ No ◯ no recall | Gemischter Wildreis ◯ Yes ◯ No ◯ no recall |
| **Boiled potatoes (daily served side dish 3)** | Salzkartoffeln ◯ Yes ◯ No ◯ no recall | Salzkartoffeln ◯ Yes ◯ No ◯ no recall | Salzkartoffeln ◯ Yes ◯ No ◯ no recall | Salzkartoffeln ◯ Yes ◯ No ◯ no recall | Salzkartoffeln ◯ Yes ◯ No ◯ no recall |
| **Salad bar (available evry day)** | Rohkost, Mariniertes o.a. Produkte der Salatbar? ◯ Yes ◯ No ◯ no recall | Rohkost, Mariniertes o.a. Produkte der Salatbar? ◯ Yes ◯ No ◯ no recall | Rohkost, Mariniertes o.a. Produkte der Salatbar? ◯ Yes ◯ No ◯ no recall | Rohkost, Mariniertes o.a. Produkte der Salatbar? ◯ Yes ◯ No ◯ no recall | Rohkost, Mariniertes o.a. Produkte der Salatbar? ◯ Yes ◯ No ◯ no recall |
| **Dessert 1** | Panna Cotta mit Beerensauce ◯ Yes ◯ No ◯ no recall | Cappuccinomousse ◯ Yes ◯ No ◯ no recall | Zitronenquark ◯ Yes ◯ No ◯ no recall | Weißweinbirne mit Schokoladen-Maronencreme ◯ Yes ◯ No ◯ no recall | Dampfnudel mit Kruste dazu Vanillesauce ◯ Yes ◯ No ◯ no recall |
| **Dessert 2** | Buttermilch-Vanillecreme mit Mandarinen ◯ Yes ◯ No ◯ no recall | Kokosmousse ◯ Yes ◯ No ◯ no recall | Bananen-Ananas-Götterspeise mit Sahne ◯ Yes ◯ No ◯ no recall | Früchtejoghurt ◯ Yes ◯ No ◯ no recall | Vanillepudding mit Schokosauce ◯ Yes ◯ No ◯ no recall |
| **Fruits salad (daily served dessert 3)** | Obstsalat ◯ Yes ◯ No ◯ no recall | Obstsalat ◯ Yes ◯ No ◯ no recall | Obstsalat ◯ Yes ◯ No ◯ no recall | Obstsalat ◯ Yes ◯ No ◯ no recall | Obstsalat ◯ Yes ◯ No ◯ no recall |
| **Backwaren (tgl. angeboten)** | Brötchen oder Scheibe Brot, pur ◯ Yes ◯ No | Brötchen oder Scheibe Brot, pur ◯ Yes ◯ No | Brötchen oder Scheibe Brot, pur ◯ Yes ◯ No | Brötchen oder Scheibe Brot, pur ◯ Yes ◯ No | Brötchen oder Scheibe Brot, pur ◯ Yes ◯ No |

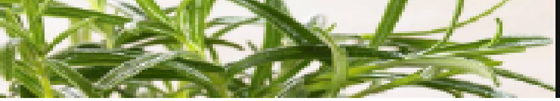

# MENÜPLAN

Modified by ROBERT KOCH INSTITU

Menüplan KFW Niederlassung Berlin Charlottenstraße

**Valid from 07.01.- 11.01.2013**

| | Monday 7.1.13 | | Tuesday 8.1.13 | | Wednesday 9.1.13 | | Thursday 10.1.13 | | Friday 11.1.13 | |
|---|---|---|---|---|---|---|---|---|---|---|
| I visited the canteen? | ◯ Yes ◯ No ◯ do not remember | | ◯ Yes ◯ No ◯ do not remember | | ◯ Yes ◯ No ◯ do not remember | | ◯ Yes ◯ No ◯ do not remember | | ◯ Yes ◯No ◯ do not remember | |
| **Maincourse 1** | Putenkeulenspieß mit Sweet-Chilli-Sauce | ◯ ja ◯ nein ◯ weiß nicht | **Bio-Eieromlette mit Champignonfüllung dazu Kräuterdip** | ◯ ja ◯ nein ◯ weiß nicht | Brandenburger Wurstgulasch | ◯ ja ◯ nein ◯ weiß nicht | Rindergeschnetzeltes Stroganoff | ◯ ja ◯ nein ◯ weiß nicht | **Bio-Currywurst (100g) mit Gewürzketchup** | ◯ ja ◯ nein ◯ weiß nicht |
| **Maincourse 2** | Bratwurstschnecke mit Sauce oder süßem Senf | ◯ ja ◯ nein ◯ weiß nicht | Paniertes Seelachsfilet mit Remouladensauce | ◯ ja ◯ nein ◯ weiß nicht | Lammhüfte gebraten dazu Sauce von roten Zwiebeln | ◯ ja ◯ nein ◯ weiß nicht | Barbarieentenbrust gebraten mit Balsamico-Honigsauce | ◯ ja ◯ nein ◯ weiß nicht | Welsfilet in Senfmarinade dazu Dillsauce | ◯ ja ◯ nein ◯ weiß nicht |
| **Maincourse 3** | 2 Kartoffeltaschen gefüllt mit Tomaten und Mozarella | ◯ ja ◯ nein ◯ weiß nicht | Gebratene Hähnchenbrust mit Käsesauce | ◯ ja ◯ nein ◯ weiß nicht | Linsenfrikadelle auf Paprika-Pilzragout | ◯ ja ◯ nein ◯ weiß nicht | Gnocchi mit gehackten Wallnusskernen ,Trüffelöl u. Weißweinsauce | ◯ ja ◯ nein ◯ weiß nicht | Raviolini mit Gemüse-füllung in Gorgonzola -sauce mit Tomaten | ◯ ja ◯ nein ◯ weiß nicht |
| **Vegetable side dish 1** | Weinsauerkraut | ◯ ja ◯ nein ◯ weiß nicht | Frische Möhren | ◯ ja ◯ nein ◯ weiß nicht | Bohnengemüse | ◯ ja ◯ nein ◯ weiß nicht | Frisches Kürbis-Karottengemüse | ◯ ja ◯ nein ◯ weiß nicht | Fenchel | ◯ ja ◯ nein ◯ weiß nicht |
| **Vegetable Side dish 2** | Brokkoli | ◯ ja ◯ nein ◯ weiß nicht | **Bio-Rosenkohl** | ◯ ja ◯ nein ◯ weiß nicht | Paprika-Pilzragout | ◯ ja ◯ nein ◯ weiß nicht | Grünkohl | ◯ ja ◯ nein ◯ weiß nicht | Rahmwirsing | ◯ ja ◯ nein ◯ weiß nicht |
| **Side dish 1** | **Bio-Spätzle** | ◯ ja ◯ nein ◯ weiß nicht | Basmatireis | ◯ ja ◯ nein ◯ weiß nicht | Spiralnudeln | ◯ ja ◯ nein ◯ weiß nicht | 4 Macairkartoffeln | ◯ ja ◯ nein ◯ weiß nicht | Hausgemachte Bio-Kartoffelwedges | ◯ ja ◯ nein ◯ weiß nicht |
| **Side dish 2** | Bratkartoffeln | ◯ ja ◯ nein ◯ weiß nicht | Lauwarmer Kartoffelsalat | ◯ ja ◯ nein ◯ weiß nicht | Rosmarinkartoffeln | ◯ ja ◯ nein ◯ weiß nicht | Polentapüree | ◯ ja ◯ nein ◯ weiß nicht | Butterreis | ◯ ja ◯ nein ◯ weiß nicht |
| **Boiled potatoes (daily served side dish 3)** | Salzkartoffeln | ◯ ja ◯ nein ◯ weiß nicht | Salzkartoffeln | ◯ ja ◯ nein ◯ weiß nicht | Salzkartoffeln | ◯ ja ◯ nein ◯ weiß nicht | Salzkartoffeln | ◯ ja ◯ nein ◯ weiß nicht | Salzkartoffeln | ◯ ja ◯ nein ◯ weiß nicht |
| **Salad bar (available evry day)** | Rohkost, Mariniertes o.a. Produkte der Salatbar? | ◯ ja ◯ nein ◯ weiß nicht | Rohkost, Mariniertes o.a. Produkte der Salatbar? | ◯ ja ◯ nein ◯ weiß nicht | Rohkost, Mariniertes o.a. Produkte der Salatbar? | ◯ ja ◯ nein ◯ weiß nicht | Rohkost, Mariniertes o.a. Produkte der Salatbar? | ◯ ja ◯ nein ◯ weiß nicht | Rohkost, Mariniertes o.a. Produkte der Salatbar? | ◯ ja ◯ nein ◯ weiß nicht |
| **Dessert 1** | Grießpudding mit Apfelmus | ◯ ja ◯ nein ◯ weiß nicht | Marzipanmousse | ◯ ja ◯ nein ◯ weiß nicht | Panna Cotta mit Anispflaumen | ◯ ja ◯ nein ◯ weiß nicht | Apfelstrudel mit Vanillesauce | ◯ ja ◯ nein ◯ weiß nicht | Haselnusspudding | ◯ ja ◯ nein ◯ weiß nicht |
| **Dessert 2** | Früchtecreme | ◯ ja ◯ nein ◯ weiß nicht | Buttermilchdessert mit Kirschen | ◯ ja ◯ nein ◯ weiß nicht | Cappuchino-Creme | ◯ ja ◯ nein ◯ weiß nicht | Mousse au chocolat | ◯ ja ◯ nein ◯ weiß nicht | Früchtejoghurt | ◯ ja ◯ nein ◯ weiß nicht |
| **Fruits salad (daily served dessert 3)** | Obstsalat | ◯ ja ◯ nein ◯ weiß nicht | Obstsalat | ◯ ja ◯ nein ◯ weiß nicht | Obstsalat | ◯ ja ◯ nein ◯ weiß nicht | Obstsalat | ◯ ja ◯ nein ◯ weiß nicht | Obstsalat | ◯ ja ◯ nein ◯ weiß nicht |
| **Backwaren (tgl. angeboten)** | Brötchen oder Scheibe Brot, | ◯ ja ◯ nein ◯ weiß nicht | Brötchen oder Scheibe Brot, | ◯ ja ◯ nein ◯ weiß nicht | Brötchen oder Scheibe Brot, | ◯ ja ◯ nein ◯ weiß nicht | Brötchen oder Scheibe Brot, | ◯ ja ◯ nein ◯ weiß nicht | Brötchen oder Scheibe Brot, | ◯ ja ◯ nein ◯ weiß nicht |
